# *In situ* organism-sediment interactions: Bioturbation and biogeochemistry in a highly depositional estuary

**DOI:** 10.1371/journal.pone.0187800

**Published:** 2017-11-27

**Authors:** S. Kersey Sturdivant, Megumi S. Shimizu

**Affiliations:** 1 INSPIRE Environmental, Newport, RI, United States of America; 2 Division of Marine Science and Conservation, Nicholas School of the Environment, Duke University, Beaufort, NC, United States of America; 3 Department of Biology and Marine Biology, University of North Carolina Wilmington (UNCW), Wilmington, NC, United States of America; Universita degli Studi di Urbino Carlo Bo, ITALY

## Abstract

Organic matter (OM) production and degradation is important in coastal estuaries, and OM fate is strongly influenced by the coupled interactions of bioturbation and biogeochemistry. From April to September 2013 sediment cores and a benthic observing system, Wormcam, were used to investigate the *in situ* relationship of biogeochemistry and macrofauna bioturbation in Cape Lookout Bight North Carolina. Wormcam imagery provided a vivid depiction of macrofauna functioning in an environment not previously observed, and affirmed the importance of fine-scale temporal observations of the benthic environment *in situ*. Observation of macrofauna presence and bioturbation during the summer contradicted previous studies that found this area to be azoic during methane activity and sulfide build-up. Sulfate concentrations decreased while sulfide and dissolved inorganic carbon concentrations increased during the summer. This coincided with changes in the depth and rates of bioturbation. Summer burrow depths (~0.8 cm) and rates (~0.4 cm h^-1^) were significantly less than spring burrow depths (~3.0 cm) and rates (~1.0 cm h^-1^). While sulfate reduction and OM degradation increased with temperature at a microscopic level, macroscopic OM degradation was reduced. As a result, reduced conditions dominated and a thin aerobic sediment layer, a few millimeters in thickness, was visible at the sediment surface. Decreases in macrofauna burrow depth and rates diminishes the area of influence of bioturbators, limiting bioturbation and subsequently the important ecosystem functions these organisms provide.

## 1. Introduction

Organic matter (OM) deposition and degradation is of critical importance in coastal estuaries given its role in biogeochemical cycling processes [1]. The fate of OM in the benthic environment is strongly influenced by the coupled interactions of sediment biogeochemistry and macrofauna bioturbation [2–4]. Bioturbation, the biological displacement or mixing of sediments [5], affects biogeochemistry through remineralization (supplying electron acceptors that fuel organic mineralization) and by increasing sediment permeability [6]. Additionally, biogeochemistry plays an important role in the depths and rates of bioturbation via the regulation of redox layers in the sediment [7,8].

In coastal estuaries bioturbation is largely driven by macrofauna [9] with vertical and horizontal mixing rates [5] that vary based on sediment grain-size, organic content/quality, community structure, and season [10–13]. The importance of macrofauna for sediment function has long been known [11,14,15] and the sediment mixed layer can serve as a proxy for benthic function [16]. Additionally, sediment properties can play a key role in structuring benthic communities [9,17,18]. In particular, macrofauna burrowing behavior has critical influence on geochemical exchanges across the sediment-water interface given the diversity and abundance of macrofauna that perforate the sediment [19,20]. However, the extent to which bioturbation and biogeochemistry influence each other is highly variable [5] and areas with high OM deposition, such as Cape Lookout Bight, NC, provide a platform to assess and quantify this relationship.

Cape Lookout Bight is a semi-enclosed coastal lagoon that experiences high deposition of organic rich material from both autochthonous and allochthonous sources [1]. Sedimentation rates in the lagoon can be as high as ~12 cm year^-1^ [21], and microbial-mediated degradation processes control the composition of OM, which varies seasonally [22,23]. As a result of the high rates of sedimentation, anoxic sediment is often observed only a few millimeters below the sediment surface during the late spring and summer months when metabolic activity is enhanced [24]. At extreme levels of organic-loading, pore-water sulfate is depleted, and methanogenesis occurs [10,25]. The process of methanogenesis is detected by the appearance of methane bubbles in the sediment column [26]. Due to the high organic matter loading throughout the year in Cape Lookout Bight, methane diffuses from the seafloor into the water column and is additionally released as bubble ebullition during the summer [27,28]; this biogeochemical activity results in seasonal variations in the geochemical zonation of the sediment (e.g. sulfate reduction zone and the methane production zone) [29].

Based on previous studies of the biogeochemical profiles of Cape Lookout Bight it was assumed that macrofauna presence and influence is minimal to non-existent [24]. Another study examining x-radiography of sediment cores showed horizontal lamination during the winter indicating a lack of bioturbation, and the sediment reworking observed in the upper sediment layer during the summer was associated with upward methane gas transport [29]. This assumption was supported by a macrofauna distribution study that found in areas of high sulfide and methane activity, macrofauna were only present from late winter to early spring (February–May). During this time a small population of spionid polychaetes (~2 cm in length) temporarily colonized the upper 3 to 4 cm of recently deposited oxidized sediment [30].

Assessments of bioturbation in the bight have not been made since the early 1980s. Since that time methodological advances have improved the ability of scientists to assess macrobenthic function [19,31]. Studies aimed at elucidating the mechanisms of bioturbation, the functional role of individual species, and the environmental barriers that shape these processes were enhanced with the development of sediment profile imaging (SPI) cameras [31]. SPI cameras utilize a prism that penetrates into the sediment to obtain undisturbed images of the upper 20 cm of the sediment column [32]. Using cross-sectional images of the sediment-water interface, studies have demonstrated the limitations of traditional macrofauna sampling. A key finding from this work was that community classification based on numerical dominance could underestimate the functional role of macrofauna communities [33,34]. Combinations of SPI and traditional core sampling offer the most robust assessment of the benthos allowing for the identification of specific species and macrofaunal functional groups, and relating this abundance and distribution data to sediment reworking behaviors and biogeochemical processes [7,8].

Traditional SPI has enhanced the understanding of sediment-organism interactions [35,36], particularly spatial changes in bioturbation relative to environmental habitat and condition [36–38]. Less well known are the fine-scale temporal dynamics of bioturbation *in situ*, a method of observation that could validate or challenge earlier assessments of the importance of macrofauna function in Cape Lookout Bight [24,29,30]. Due to the difficulty of *in situ* observation of the sediment-water interface, fine-scale (on the order of hours) temporal observations have been limited to only a few days or weeks [19,32,39]. One study to date has made fine-scale temporal assessments over multiple seasons [8]. Using a benthic observing system called Wormcam, Sturdivant et al. [8] examined traditional paradigms regarding macrofauna function during anthropogenic disturbance, and established the importance of long-term, fine-scale, *in situ* study of benthic systems. In our study we used Wormcam to assess macrofauna presence and bioturbation in Cape Lookout Bight relative to sediment biogeochemistry. Presence was determined by active biogenic structures as no abundance data was collected. The specific objectives of our study were to 1) assess the temporal presence of macrofauna in Cape Lookout Bight, 2) quantify macrobenthic function through burrowing rates and the maximum depth of bioturbation, and 3) assess the relationship between macrobenthic function and sediment biogeochemistry through rates of sedimentation and optical geochemistry patterns.

## 2. Methods

### 2.1 Study Area

**Fig 1:**
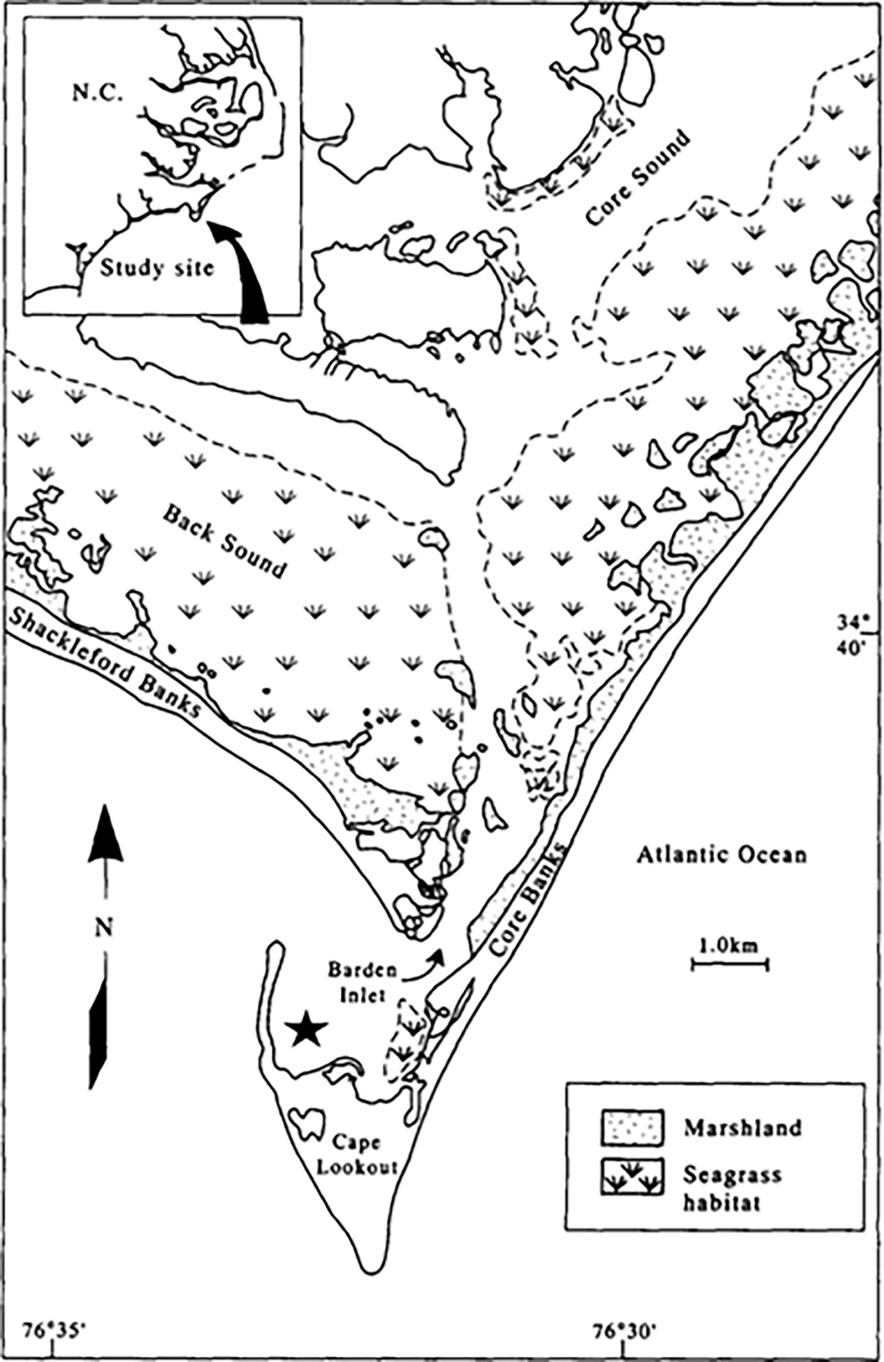
Study area of Cape Lookout Bight estuary. The black star represents the approximate location of the study site.

Cape Lookout Bight (Fig 1) is a 2 km^2^ area coastal lagoon located southeast of Cape Hatteras in the outer banks of North Carolina (34°37’N, 76°33’W). The bight waters in the semi-enclosed basin are influenced by both the back barrier island lagoon system as well as water from offshore [21]. The geographical characteristics of the bight help to hold fine-grained sediments that are mostly constituted of silts and clays, and organic debris from sounds through Barden Inlet [40]. The surficial sediments can be primarily black mud and contain about 4% organic carbon [24]. Sediment and OM accumulation is higher in early spring to summer (February to June) and associated with storm events, while less accumulation occurs during fall to winter [22]. No specific permission was required to sample in Cape Lookout Bight as the area is not a state or federally protected area. Additionally, the field studies did not involve endangered or protected species.

### 2.2 Sediment biogeochemistry sampling

Sediment cores were collected in April, June and August of 2013, using a piston core, close to the study location of Martens et al. [29]. Sediment cores were kept vertical during recovery until processing, where cores were extruded and sectioned every 2 cm for the first 40 cm, and every 5 cm for the rest of the core (maximum core length 100 cm). Duplicate sediment cores were collected for comparison and all cores were processed within 6 hours after recovery.

### 2.3 Geochemical pore water analysis

Sediment pore water was collected by centrifugal extraction and filtered with a 0.2 μm filter. Sulfide concentrations in porewater was preserved in 20% Zn acetate solution and analyzed with the spectro-photometric method [41]. Sulfate concentrations were determined following Tabatabai [42]. Dissolved inorganic carbon (DIC) concentrations were measured in triplicate by acidification and subsequent quantification of released CO_2_ using a LiCor 7000 CO_2_ detector [43]. The measurements were calibrated against certified reference materials purchased from Scripps Institution of Oceanography, University of California San Diego.

### 2.4 Wormcam

The Wormcam system similar to Sturdivant et al. [8] was used in this study. It consisted of an IQEye model 705 5-megapixel Ethernet camera, placed in a plastic housing with a 45-degree angle at the bottom, which formed a wedge to penetrate into the sediment. A front surface mirror on the back wall in the wedge reflected the sediment profile to the camera. The field of view (FOV) was 12 cm wide by 18 cm deep. Lighting was provided by a single white LED (Lexeon Star model 5C). The camera was set to take a burst of 5 images about 10-seconds apart every hour, which were stored on a memory card. The 5 images were collected as quality control “replicates” to ensure a “best” image was captured for each specific time point. Wormcam was affixed to a low-profile PVC frame to minimize flow disturbance and to prevent the camera from fully sinking in the sediment [8]. The Plexiglas faceplate extended beyond the edges of the housing to prevent eddy-induced erosion near the FOV. A Hach DS500X water-quality datasonde was attached to the frame 20 cm above the sediment-water interface and collected DO, salinity, temperature, and depth measurements at 1 hr intervals synchronous with image capture. The system was controlled by a Macromatic Series C TR-65126 Time Delay Relay and was powered from a 12-V battery in a separate housing connected by cable to the Wormcam prism [8]. During maintenance trips the memory card was retrieved and images were downloaded for analysis of biogenic structures and sediment properties, and the water-quality datasonde was replaced. Wormcam was deployed for 4 ½ months, from mid-May to September at ~9 m depth (Table 1). Maintenance was conducted every 3–4 weeks to reset the system and change the batteries and WQ sonde. The system was reset (i.e., retrieved for maintenance and subsequently redeployed in approximately the same GPS location) to account for the facilitation of burrow formation at the sediment-faceplate interface, which over time attracts larger organisms, artificially restructuring the benthic community in front of the faceplate [44].

**Table 1:** Sampling dates and locations.

Date	Sample Type	Latitude	Longitude
16 April 2013	Piston core 1	34°37'5"N	76°32'53"W
16 April 2013	Piston core 2	34°37'6"N	76°32'53"W
15 May 2013	Wormcam deployment	34°37'5"N	76°32'54"W
12 June 2013	Piston core 1	34°37'5"N	76°32'50"W
12 June 2013	Piston core 2	34°37'6"N	76°32'51"W
12 June 2013	Wormcam reset	34°37'5"N	76°32'51"W
10 July 2013	Wormcam reset	34°37'5"N	76°32'53"W
06 August 2013	Wormcam reset	34°37'5"N	76°32'52"W
13 August 2013	Piston core 1	34°37'6"N	76°32'54"W
13 August 2013	Piston core 2	34°37'5"N	76°32'56"W
06 September 2013	Wormcam retrieval	34°37'5"N	76°32'52"W

### 2.5 Image analysis

Photoshop (Adobe Systems Inc.) was used to rotate and scale images, and ImageJ (NIH) was used for digital measurements of sediment properties and biogenic structures. Quality control on image data was accomplished by reanalysis of a random selection of 25% of the images by a second analyst. A 5 hr interval, totaling 547 images, was used to comparatively assess macrofauna activity and sediment properties. Oxidation state of the sediment and depth of the apparent-color redox-potential discontinuity (aRPD) was determined by color: tan-brown sediment was considered oxidized and grayish-black sediment was considered reduced [45]. During deposition events the “old” sediment surface initially retained its oxic properties, but overtime would return to subsurface coloration. Legacy surface layers were ignored during aRPD measurement, and the depth from the surface to the new aRPD was used in measurements. Burrow depth was the maximum depth that biogenic structures, burrows or feeding voids, were detected. Sediment grain-size major mode and range were visually estimated at a 5 hr interval, by overlaying a grain-size comparator at the same scale as the image [46]. The grain-size comparator is a proprietary tool used to determine sediment grain size in sediment profile images (SPI). This comparator was prepared by photographing a series of Udden-Wentworth size classes (equal to or less than coarse silt up to granule and larger sizes) with the SPI camera. Seven grain-size classes were on this comparator: >4 ϕ (silt-clay), 4–3 ϕ (very fine sand), 3–2 ϕ (fine sand), 2–1 ϕ (medium sand), 1–0 ϕ (coarse sand), 0-(-1) ϕ (very coarse sand), <-1 ϕ (granule and larger). The diameter of larger grain-sizes was measured to determine their size class. The lower limit of optical resolution of the SPI system was about 62 microns, allowing recognition of grain sizes equal to or greater than coarse silt (>4 ϕ). Rates of deposition and accretion were measured as the change in sediment height from the preceding image, and for analysis were represented as the absolute change in sediment height. Whenever the system was reset during maintenance, the very first image captured after redeployment did not contain a sediment height calculation as there was no preceding image to compare with. However, the next image captured was compared to its preceding image, and so-forth until the system was reset. Replicate measures of the change in bed height excluded fecal mound development and burrow entrances. Each factor assessed (e.g. aRPD depth, burrow depth, change in sediment height) was measured 3 times per image, providing a mean and standard deviation (SD) for each image. Each image was divided into 3 sections and a random point in each section was used for the location of measurement. This approach allowed for the accurate capture of localized variability observed in some SPI. Burrowing morphology and sediment properties were recorded from a total of 547 images. The relationships between sediment biogeochemistry and biogenic data were assessed by regression analysis.

To determine burrow lengths and rates, 88 individual burrows were followed through time at 1 hr intervals. These individual burrows were assigned a unique ID and the burrow shape, burrow depth, burrow length, burrow duration (time burrow was actively inhabited), and cause of vanishing were all recorded. Burrow rates were recorded and defined as the plus or minus change in burrow length per hour, and accounted for the burrowing activity of worms. The shape and form of a burrow can be important in species identification. Burrow shape was classified in three categories: 1) U-shaped–burrows were connected with two openings at the sediment water interface (e.g., *Polydora tetrabranchia*); 2) straight–burrows that are relatively straight in form (e.g., *Glycera* spp.); and 3) curved–burrows had at least one meander (e.g., *Heteromastus filiformis*). When assessing burrow lengths from SPI it was accepted that there may have been unknown confounding variance due to the two-dimensional assessment (the profile image) of a three-dimensional structure (the sediment column). If animals were burrowing away from the faceplate the length of burrows could have been underestimated. To limit the potential for this confounding factor in burrow length measurements, only those burrows with a distinct connection to the sediment surface were included in calculations.

### 2.6 Data analysis

Correlations between sediment geochemistry quantified from SPI (aRPD depth) and infauna bioturbation (burrow depth and burrow length) were tested. Because of the experimental design, the residuals were not assumed to be random, (i.e. independent variables drawn from a normal (0, sigma) distribution. The residuals for each regression were plotted against time to determine if a pattern existed or they were randomly distributed. If a pattern existed, the autocorrelation structure would have been accounted for in the estimation of the regression coefficients. However, no pattern of autocorrelation existed for the tested variables, and general linear models were employed. To assess the variability in sediment geochemistry (aRPD depth), and bioturbation (burrow depth), Kruskal-Wallis H test, a non-parametric analysis of variance (ANOVA), was conducted. Dunn’s test, a pairwise comparison following ranked-based ANOVA, was performed to assess significant differences. Non-linear regression was used to assess the relationship between bioturbation (represented by burrowing rates) and rates of deposition, where an exponential curve was fit to the data. For all analyses normality was checked with Shapiro-Wilk’s test, and homogeneity of variance was assessed with Bartlett’s test [47]. All of the raw data used in this study has been made available [S1 File].

## 3. Results

### 3.1 Physical environment

Our study period was a 5-month interval from April to September 2013. Mean bottom water temperature was 25 ^o^C (SD ±5), mean salinity was 35 psu (SD ±3), and mean DO concentration was 5.8 mg l^-1^ (SD ±1.1). The sediment grain-size major mode was >4 phi, which represents fine silt-clay mud sediments typical of organic-rich high deposition areas.

### 3.2 Macrofauna

**Fig 2:**
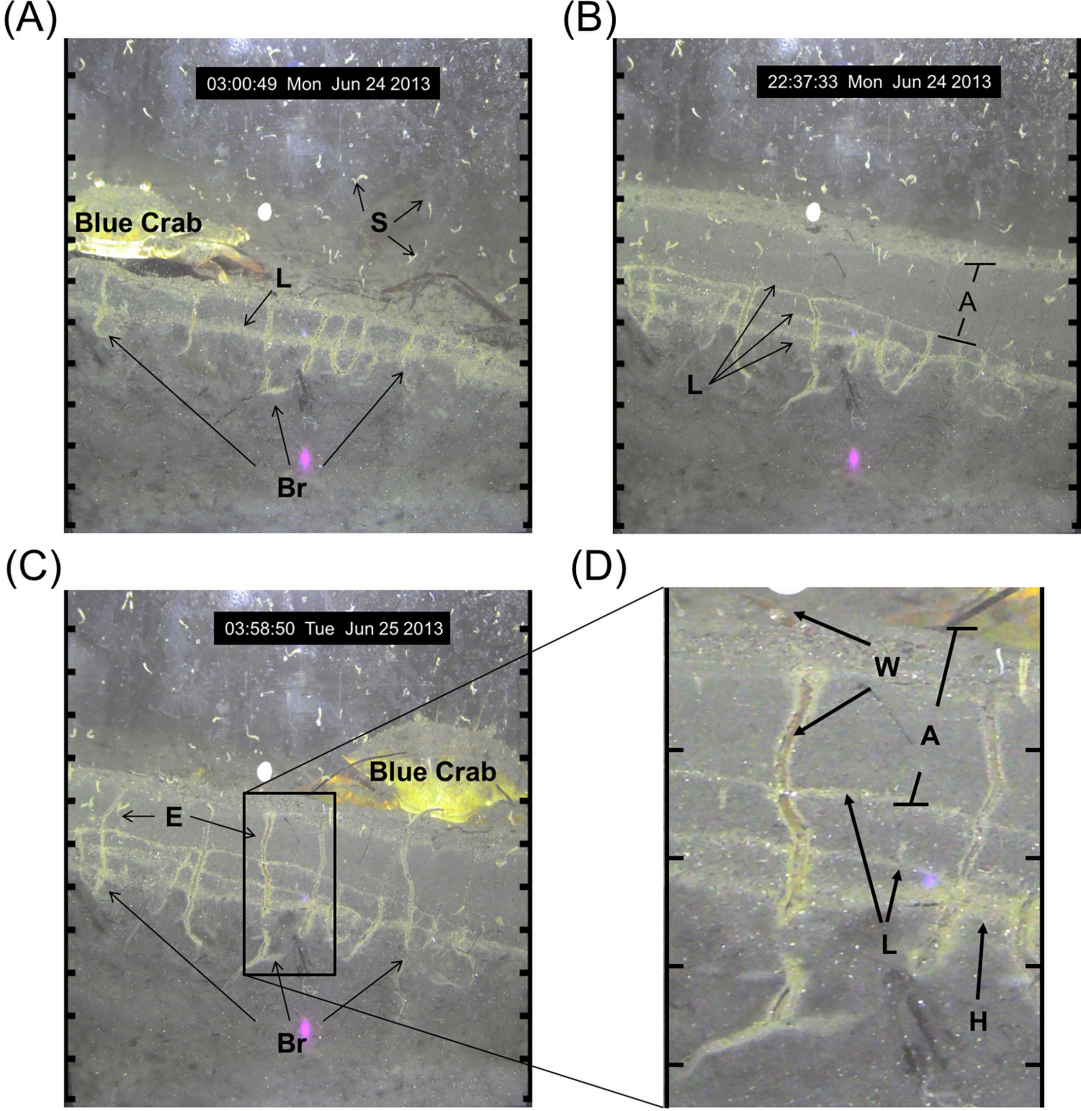
Representation of sediment dynamics in Cape Lookout Bight. (A) Sediment profile showing oxidized U-shaped *Polydora tetrabranchia* burrows [Br], a legacy sediment surface layer [L], and *P*. *tetrabranchia* larvae recruiting on the face plate of the prism [S]. (B) An approximately 4 cm accretion of sediment [A]. (C) *P*. *tetrabranchia* extending their burrows [E] back to the surface in response to the accretion event. (D) Magnification of a U-shaped burrow, with a visible *P*. *tetrabranchia* in its burrow and extending a portion of its body out of the burrow [W] and the oxic halo [H] produced from burrows into the surrounding reduced sediment. Scale around images is in cm units. Light artifacts from reflection in the prism visible at the bottom (purple color) and top (white color) of images.

Image analysis showed macrofauna burrowing throughout the 4.5-month observation period. The primary bioturbator was the spionid, *Polydora tetrabranchia*, identified by its unique U-shaped burrows (Fig 2), morphological features, and recruitment strategies [48,49]. Greater than 50% of all burrows observed were U-shaped, indicating the dominance of *P*. *tetrabranchia* in this system; 38% of burrows were straight, and 10% were curved.

### 3.3 Geochemical profiles

**Fig 3:**
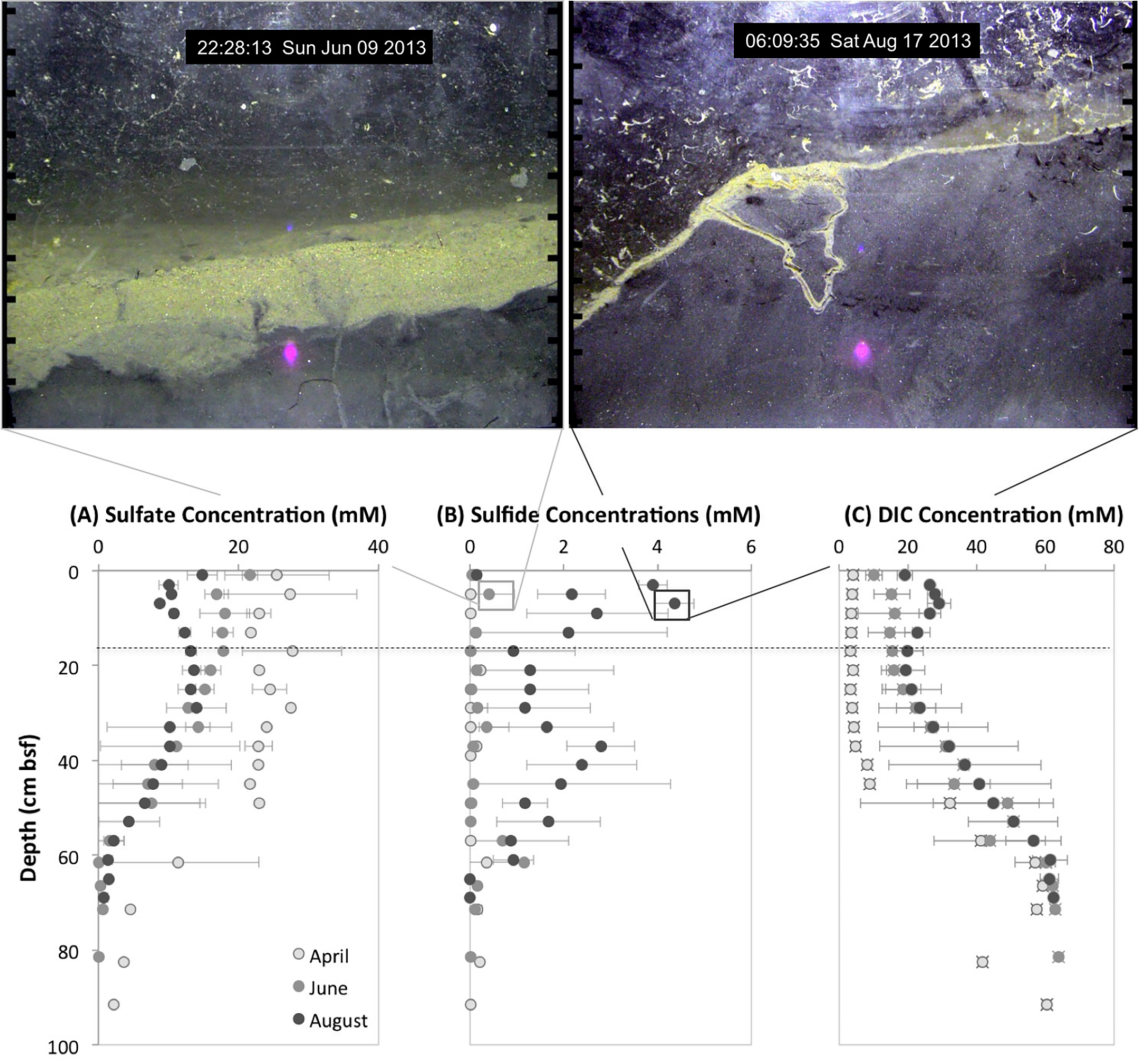
Geochemical depth profiles of Cape Lookout Bight sediments. Depth profiles in April, June and August of (A) sulfate, (B) sulfide, and (C) DIC concentrations. Black horizontal dashed line represents maximum vertical FOV of Wormcam. Error bars represent ±1SD. (bsf: below seafloor). Representative sediment profile images for June and August are shown above graphs. Scale around images is in cm units. Light artifact from reflection in the prism visible at the bottom of images (purple color).

To characterize the spring-summer changes in the geochemical profiles of sediment porewater, sulfate, sulfide, and dissolved inorganic carbon (DIC) concentrations were measured. In the top 10 cm of the sediment we found decreases in sulfate concentrations and increases in sulfide and DIC concentrations from April to August (Fig 3). These results suggest that from April to August the sulfate reduction rate and OM degradation rates within the sediment increased as water temperatures increased. In April, in the top 10 cm of the sediment, sulfate concentrations ranged between 20 to 30 mM and decreased to < 5 mM at 90 cm depth (Fig 3A). This reduction was most likely due to microbial sulfate reduction (e.g. *Desulfovibrio* spp.) as sediment DIC increased inversely with sulfate reduction [50]. In June and August, sulfate was reduced in the surface sediments compared to April, and completely depleted by 60 cm depth. Sulfide concentrations in April cores were below 0.5 mM, and in June below 1.5 mM; both cores had their highest sulfide concentrations at 60 cm depth. In August, sulfide concentrations ranged between 1 to 4.5 mM above 60 cm depth (Fig 3B). Together with sulfate profiles, our results indicate that sulfate reduction rates in the sediments were higher in August compared to April and June. DIC concentrations in the cores were lowest in April (3 to 9 mM above 60 cm depth), and highest in August (20 to 50 mM above 60 cm depth; Fig 3C). In June, DIC concentrations were close to the profile in August and ranged between 10 to 50 mM in the top 60 cm.

### 3.4 Bioturbation

**Fig 4:**
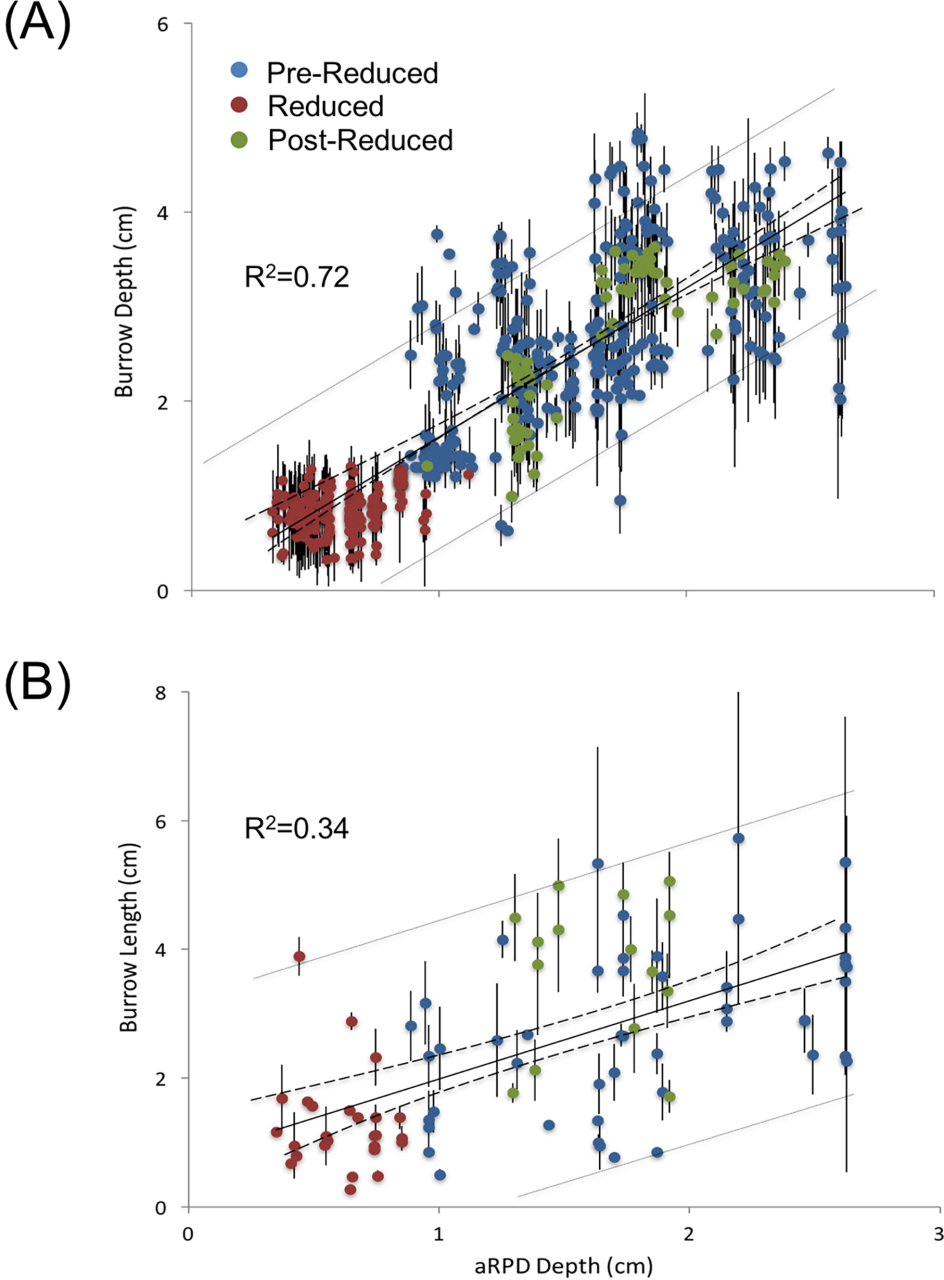
Relationship between aRPD depth and bioturbation (burrow depth and burrow length). A significant positive relationship was found between aRPD depth and (A) burrow depth (df = 546, F = 1414.1, p<0.001) and (B) burrow length (df = 87, F = 27.3, p<0.001). Error bars represent ±1SD. Dashed lines represent regression confidence interval (CI), and lighter dotted lines represent population CI.

During our observation period burrowing activity was detected within the first hour of deployment of the Wormcam system. Small spionid-like worms in U-shaped burrows were observed above the aRPD near the sediment-water interface (SWI). On average the initial burrow lengths were ≥ 70% of the maximum length (i.e., the largest measured length of the burrow during our observation period), indicating that the majority of burrow formation was completed within an hour–the time interval between images. The initial length of less than a quarter of the burrows were ≥ 90% of the maximum length. Burrow morphology was significantly correlated with aRPD depth, which is a boundary proxy for changes in sediment biogeochemistry that can be influenced by burrowing activity [35]. Burrow depths (Fig 4A) and burrow lengths (Fig 4B) were significantly positively correlated to aRPD depth. Increases in burrow length occurred primarily from burrows extending into newly accreted sediment; worms would extend their burrows back to the surface within an hour following high accretion events (Fig 2). Worms were occasionally observed burrowing deeper during erosion events. The aRPD explained more of the variability in burrow depths than burrow lengths, likely due to the variability in burrow type. Fauna have different burrowing strategies, and the burrow types in our study were variable; 38% of the macrofauna in our study had straight burrows. The rest of the burrows (72%) were U-shaped or curved. Non-straight burrows (i.e. U-shaped or curved) are not specifically oriented with the vertical orientation of the sediment biogeochemistry and thus may not be as strongly correlated with aRPD depth.

Burrows generally had two distinct sections, the portion of the burrow above the aRPD and the portion below the aRPD. When assessing burrow and sediment dynamics, the aRPD is used as a delineating demarcation. In our study 65% of the burrows analyzed extended from the surface to below the aRPD. On average burrows extended 1.0 cm (SD = 0.6 cm) below the aRPD. Worms did not appear to favor either side of the aRPD and were observed moving throughout the vertical extent of their burrows during all conditions. Of these burrows 100% were oxic above the aRPD, and 98% were oxic below the aRPD. Burrows that had oxic portions below the aRPD produced an “oxic halo” along the length of the burrow below the aRPD, which extended from the burrow walls into the reduced sediment on average 0.2 cm (SD = 0.06 cm).

**Fig 5:**
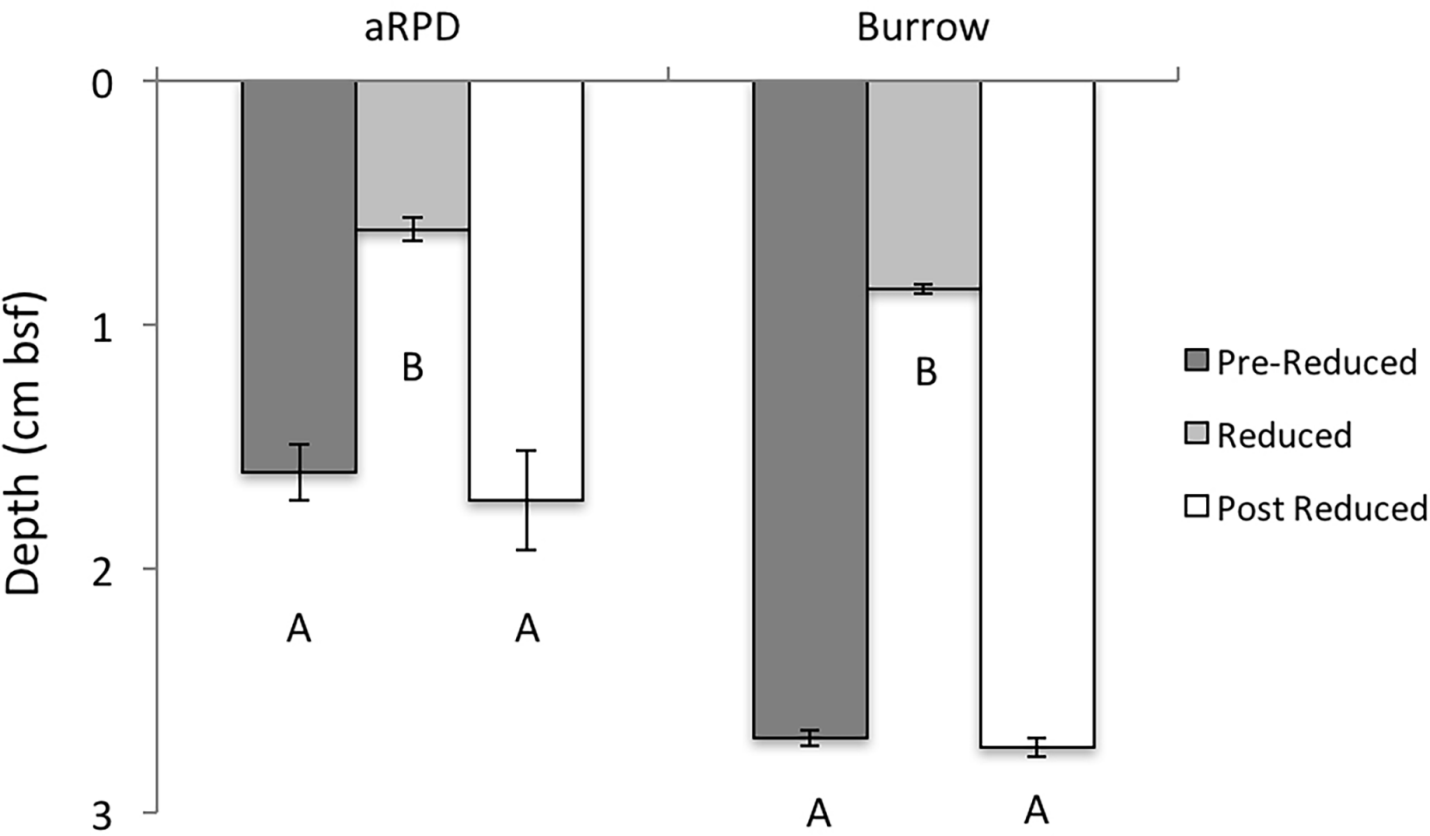
Assessment of sediment and bioturbation properties. aRPD and burrow depths during the 3 designated periods (Pre-Reduced, Reduced, Post Reduced) during the 4 ½ month study. There was a significant difference in the aRPD depths (df = 2, H = 383.03, p<0.001) and burrow depths (df = 2, H = 370.9, p<0.001) between our 3 periods; aRPD depths and burrow depths were significantly lower during the reduced period. Error bars represent ±1SE. (bsf: below seafloor).

We found visual analysis of the sediment biogeochemistry matched the biogeochemistry trends in our core samples. From the SPI we determined 3 distinct temporal periods, pre-reduced, reduced, and post-reduced periods of sediment biogeochemistry. The major sedimentary biogeochemical change occurred during the mid-summer (07/15/2013–08/24/2013) when the sulfate reduction zone was just below the sediment surface (Fig 3). This period was optically identified by an increase in sediment oxygen demand (dark black sediment) and expansion of the sulfate reduction zone (thin oxic layer) and was designated as the “reduced period”; the time periods before and after this event were noted as the pre-reduced period (05/20/2013–07/14/2013), and post-reduced period (08/24/2013–09/10/2013). We examined variations in sediment dynamics and burrowing behavior in the three periods. Burrow and aRPD depths were significantly smaller during the reduced period, compared to before and after (Fig 5).

**Fig 6:**
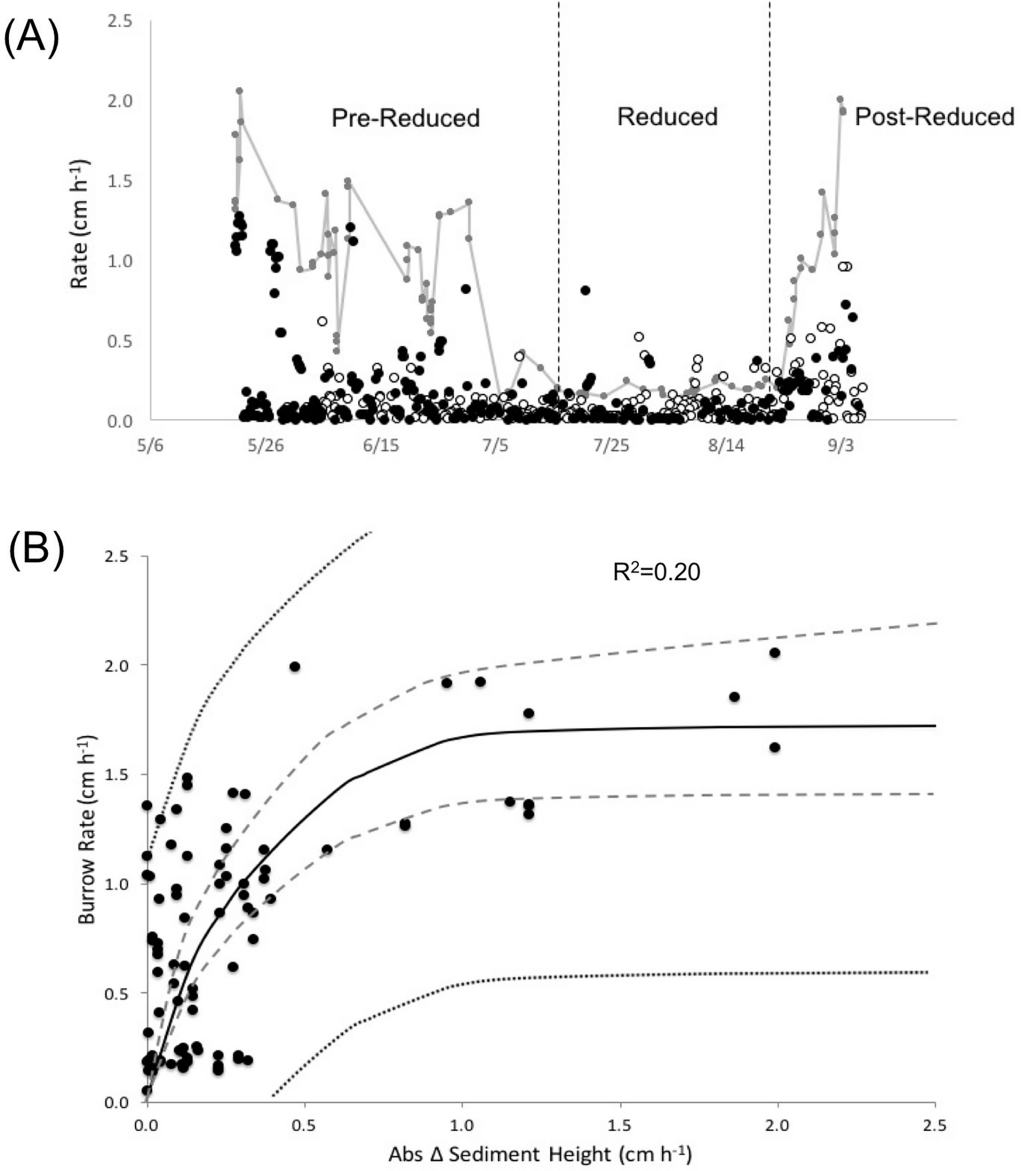
Burrow rates and sediment deposition. (A) Timeline of sediment deposition (black circles), sediment erosion (white circles), and infauna burrowing (gray circles and line) rates during the study period. Vertical dashed line delineates the 3 sediment periods. (B) Relationship between infauna burrowing rate and sediment deposition and accretion events (represented by the absolute change in sediment height). A significant positive relationship was found between burrow rates and the change in sediment height (df = 88, F = 21.8, p<0.0001). Dashed lines represent the regression 95% confidence interval, and dotted lines represent the 95% prediction interval.

Rates of burrow production were significantly lower (df = 2, H = 184.3, p<0.001) during the reduced period (0.2 cm h^-1^) compared to the pre-reduced (1.0 cm h^-1^) and post-reduced (0.9 cm h^-1^) periods (Fig 6A). The discrepancy in burrowing rates during the reduced period was largely due to sediment deposition and accretion, assessed as the absolute change in sediment height (cm h^-1^). Infauna burrowing rates significantly correlated with both the timing and magnitude of erosion and accretion events (Fig 6), with burrowing rates reaching an asymptote just below 2.0 cm h^-1^, regardless of the continued increase in sediment change from erosion or accretion.

## 4. Discussion

### 4.1 Macrofauna presence in Cape Lookout Bight

Macrofauna have an established presence in Cape Lookout Bight and a measurable impact on the benthic ecosystem system via bioturbation. Earlier assessments of the bight found that the area was primarily void of macrofauna, except in winter and early spring [24,29,30]. Our findings are not consistent with previous observations. However, our analyses suggest that the abiotic dynamics of this benthic system have not drastically changed from the observations made in earlier studies. The bight still experiences high rates of sedimentation ([21]; Fig 2), anoxic sediments are still observed a few millimeters below the sediment surface ([24]; Figs 2 and 3), and there are still seasonal variations in the geochemical zonation of the sediment ([24]; Fig 3). This supports our hypothesis that methodological limitations may have been a factor in underestimating the functional importance of macrofauna in the bight. Historically, macrofauna presence in CLB was assessed by inference on X-radiographs [24] and with a seasonal macrofauna distribution study [30]. In contrast, the power of SPI is the undisturbed, vivid depiction, provided of the upper 20 cm of the sediment column [46], and Wormcam allowed us to expand those observations at a fine temporal scale (observations on the order of hours) over an extended time frame (study period of 4 ½ months).

Our image analysis, and identification of macrofauna from previous studies in the area [30,51], suggest the spionid worm, *Polydora tetrabranchia* [48] was responsible for the majority of burrowing activity observed. *Polydora* spp. polychaetes form U-shaped burrows and are surface deposit and suspension feeders [49]. The dominant presence of *P*. *tetrabranchia* supports the idea of the bight being a disturbed benthic system. Spionids are highly adaptive species with the ability of both lecithotrophic and planktotrophic larval recruitment [52]. The method of nutritional recruitment has consequences for dispersal distance and population size [53,54], though neither nutritional strategy guarantees increased settlement success [55]. Macrofauna larval recruitment is selective [56,57], and the adaptability of spionid reproductive strategy increases the probability for successful larval settlement and population resiliency [52,58]. Reproductive strategies play a major role in dynamics of the population and continuity of macroinvertebrates [59], especially in disturbed systems like Cape Lookout Bight.

The bight is a dynamic estuary with sedimentation rates that can be as high as ~12 cm year^-1^ [21]. Hydrodynamic conditions that produce periods of high sedimentation increase the benthic boundary layer, favoring suspension-feeding organisms [60–62]. Once *P*. *tetrabranchia* has recruited, the probability for survival is enhanced by the adaptive feeding strategies of these polychaetes; spionids can selectively switch between suspension and deposit feeding [49,59,63]. When flow exceeds the erosion velocity spionid polychaetes switch from surface deposit feeding to suspension feeding, capitalizing on the suspended food particles in the benthic boundary layer [63]. Switching the feeding method is a key life history strategy for polychaetes in hydrodynamically active areas such as the bight, where periods of increased flow or decreased flow might leave exclusive deposit or suspension feeders nutritionally bereft. Suspension feeding is a more energetically efficient strategy for spionids [64], so periods of high flow would benefit *P*. *tetrabranchia* growth in relation to periods of low flow. Thus, it is not surprising that the spionid *P*. *tetrabranchia* is the dominant species given its eurytopic life history strategies and known resilience in areas that might be uninhabitable by more sensitive taxa [65,66].

### 4.2 Biogeochemistry in Cape Lookout Bight

The general temporal trend observed in the sediment biogeochemistry of Cape Lookout Bight during our study was unchanged from what was described previously: in the upper sediment column (< 20 cm) sulfate concentrations decreased and sulfide concentrations increased from spring to summer [27]. This reduction was most likely due to microbial sulfate reduction (e.g. *Desulfovibrio* spp.) as sediment DIC increased inversely with sulfate reduction [50]; increased sediment DIC concentrations indicate higher respiration rates and microbial activity. However, we found dissimilarities in the depth of the sulfate reduction zone, which expanded much deeper in our sediment cores than what had been documented previously. In the sediment cores collected in August 1983 and in July 1990, sulfate was depleted by 8 to 20 cm depth [27,28]. In our sediment cores collected in August 2013, the sulfate reduction zone expanded to 60 cm depth, a substantial difference. The discrepancy in sulfate reduction depth is likely the result of variations in rates of organic matter deposition and/or oxygen penetration depths [1], or may reflect changes in geochemical properties of the sediments related to larger scale changes in the region over the past 30 years [21].

The source of sulfide is sulfate reduction [67], and increased sediment microbial activity correlated with increased temperatures is the likely culprit for the high concentrations of sulfide in the bight sediments in August [67]. The concentrations of sulfide observed were as high as 4 mM, but not as extreme as some other environments. To put these concentrations into perspective, the sulfide concentrations observed in August 2013 are higher than what is documented in tropical seagrass beds (< 2mM) [68] and in temperate coastal sediment (< 0.2 mM) [69,70]. The concentrations are similar to what is normally observed in the surface sediments (upper 10 cm) of cold seeps covered by microbial mats (1–5 mM), but are lower than in the sulfate-methane transition zone (usually deeper than 50 cm) with maximum sulfide concentrations of 10–12 mM [71,72].

During the summer, the sediment of Cape Lookout Bight is a unique system. High organic matter input results in sulfate depletion near the sediment surface and results in a severely reduced sedimentary environment where high concentrations of sulfide reside near the surface [25]. Despite the high concentrations of sulfide in surface sediments there was still burrowing activity, though the depth and rates of that activity decreased when sulfide concentrations were at their highest levels in late July–mid-August.

Hydrogen sulfide is generally a highly toxic substance [73]. Its best-known and most drastic effect is disruption of terminal electron acceptance by binding to cytochrome aa_3_, resulting in the inhibition of aerobic respiration [74,75]. However, spionid polychaetes have documented resiliency to sulfide toxicity [65], though the exact mechanism by which they are able to tolerate exposure to sulfide is unknown. No sulfide-resistant cytochrome c oxidase has ever been demonstrated [76,77], but aquatic organisms have evolved various adaptations against sulfide toxicity [78].

In the natural environment chemical and biological processes can reduce/remove sulfide concentrations produced in the sediments and macrofauna organisms benefit from association with these areas [8]. Sulfide-oxidation by bacteria has been documented in organic rich coastal sediments [8,70,79], and is also well known in symbiotic invertebrates (e.g., tubeworms, mussels and clams; [80]), bacterial mats in deep-sea hydrothermal vents [81,82], and in cold-seeps [83]. It is very likely that the increased bottom water DO levels present within and around burrow structures played a facilitating role in macrofauna resilience to high sulfides in the sediment column [84].

Bottom water oxygen saturations were normoxic throughout our study, and burrows with connections to the sediment surface were fully oxidized. This oxygenation allows for oscillations between oxic and anoxic conditions inside of burrows. Cyclical redox oscillation is common within individual burrow structures and is accompanied by rapid switching in dominant metabolic processes [84]. As a result, though sediment conditions outside of the burrow may be toxic due to reduced sediments with high concentrations of sulfide and low pH [85], normoxic bottom water oxygen saturations and burrow ventilation can assist in buffering worms from the adverse sediment conditions surrounding their burrows [84,86].

### 4.3 Coupled interactions of biogeochemistry and bioturbation

Despite the ability of worms to survive the harsh sediment conditions of the bight, bioturbation was significantly limited during periods of reduced sediment biogeochemistry, as indicated by the relationships between aRPD depth and burrow depths and lengths. The aRPD depth, the boundary layer between aerobic and anaerobic sediments [7], is an effective proxy for sediment biogeochemistry as its position has fundamental consequences for biota [85]; the reduced sediments below the aRPD tend to be toxic to macrofauna due to free hydrogen sulfide and low pH [87], however many macrofauna have developed adaptive strategies (e.g., irrigate burrows) to help resist the negative impact associated with reduced sediment [15,46].

Using what we know from our sediment cores and image analysis, we are able to hypothesize a mechanism by which macrofauna function was perturbed and limited in Cape Lookout Bight. As the seasons progressed and temperatures increased, microbial activity was enhanced contributing to expanded reduced conditions in the sediment (Fig 3C). This can be seen in a comparison of sediment profiles from the spring and summer. Oxidized ferric iron gives the surface sediment the tan-brown color ([45,88]; Fig 2A), and evidence of organic enrichment (and subsequently sediment reduction) is indicated by the presence of subsurface methane gas or dark sulfidic sediment ([26,88]; Fig 2B). From our core sampling we observed sulfide concentrations to be four times higher during this summer-reduced period compared with earlier in the spring. The increases in reduced conditions in surface sediments was also documented in our visual analysis where a significantly shallower aRPD from late July to mid-August existed; decreases in the aRPD coincided with shallower burrow depths and smaller burrow lengths (Fig 4) which suggests an inhibition in bioturbation [8]. So why is this important?

A reduction in burrow depths and lengths diminishes the area of influence of bioturbators [8], limiting bioturbation by decreasing the biologically active zone (BAZ) within the sediment. The BAZ extends from the surface down into the sediment to the maximum depth of subsurface biogenic structures [89]; the primary biogenic structures used to define the BAZ are burrows and feeding voids [31]. When the BAZ decreases, ecosystem function is stymied by limiting the amount of sediment reworked [15,86]. The consequences of inhibiting bioturbation are likely to cascade to a variety of physical, biological and chemical processes, including organic matter remineralization and decomposition, nutrient cycling, pollutant release, sediment resuspension, and microbial activity [90]. While the BAZ is used as a proxy for benthic community dynamics and ecosystem processes, and provides a reasonable approximation of benthic ecosystem functioning, the findings are context-dependent (e.g. faunal mixing, food input, environmental conditions; [16]).

Decreases in burrow depths and lengths during the summer when the sulfate reduction zone was shallower was to be expected, but the decrease in the rate at which sediment was reworked suggests a macrofauna response to increased sediment toxicity at either an individual or community level. For example, the fitness of organisms could have decreased accounting for the reduction in burrowing activity, or the community could have shifted to smaller organisms due to the toxic disturbance; a common occurrence in disturbed systems [87,91]. The rate of sediment reworked was 80% lower during the summer-reduced period, limiting the biodiffusive and bioadvective components of bioturbation. Macrofauna biodiffusion and bioadvection is important for increasing the quality of marine sediments, allowing microbial oxidizers to flourish and process reduced compounds [92]. Further, burrows connected to the sediment surface, even after abandonment, act as conduits for the bioadvection of reduced compounds out of the sediment and into the water column [13,86]. The longer and deeper these burrows weave into the sedimentary fabric, the greater influence they can have on diagenetic processes [19,86,92]. The decrease in burrow rates during the summer also correlated with the rate of deposition and erosion observed in the bight. There was a noticeable decrease in the magnitude of sediment deposition and erosion during the summer reduced period when infauna burrowing rates were at their lowest. However, deposition and erosion events only accounted for 20% of the variation in infauna burrowing rates (Fig 6B) suggesting other factors had a greater influence on rates of bioturbation. It’s possible the disturbance from this reduced period changed the complexity of the infauna community. During periods of disturbance benthic successional theory suggests smaller infauna with lower rates of bioturbation are present [93]. On a few occasions during the summer reduced period rates of erosion and deposition increased, while burrowing rates did not respond; this is dissimilar to the trends observed during the pre-reduced and post-reduced periods (Fig 6).

During our observation period burrow depths at Cape Lookout Bight were relatively shallow, averaging less than 3 cm in depth during the non-reduced periods, and less than 1 cm in depth during the reduced period. The depth and type of burrows provides context about the state of the benthic community as represented by successional stage [93]. Changes in the composition of benthic infaunal assemblages can reflect the extent of organic enrichment (pollution) or physical stress encountered by the resident infauna [46], such that a predictable and sequential appearance of particular faunal traits or functional types, rather than a number of named species, occurs following environmental disturbance [93,94]. The theory of functional succession in sediment communities is typically represented by 3 qualitatively distinct successional stages [93,94]. Highly disturbed systems exhibit Stage 1 fauna represented by small tubicolous surface infauna. The contrast is an undisturbed system where Stage 3 taxa are present, represented by deep burrowing, large-bodied infauna, resulting in distinctive subsurface excavations (i.e., feeding voids). The lack of feeding voids in our images, and the presence of shallow burrows along with small surface tubes (Fig 2) indicates that the benthic community in Cape Lookout Bight is distinctly at a Stage 2 succession. Though there are significant seasonal differences in the burrowing depths and rates at Cape Lookout Bight, the differences are somewhat relative as characterization of the sediment column indicates that from Spring to Fall the sediment community in Cape Lookout Bight does not largely change from Stage 2. The characterization of Cape Lookout Bight at this successional stage supports what we know about the seasonal dynamics of the bight [24]. The high loads of nutrient input promote reduced conditions in the sediment column [22,24], but other important water quality variables remain healthy (e.g. bottom water DO concentrations were normoxic). The unique dynamics of this ecosystem appear to promote a benthic community that undergoes periodic disturbances, at least from spring to fall.

Though it is clear sediment biogeochemistry limited macrofauna function in CLB, biogeochemistry and bioturbation are coupled interactions in the sediment [95]. The presence of macrofauna bioturbators during the summer-reduced period indicates that many of the bioturbative processes discussed earlier were still occurring despite the decrease in macrofauna influence. Where bioturbators do not exist gases diffuse only a couple of millimeters into the sediment and the RPD is positioned just at or below the sediment surface [96]. The majority of burrows in CLB were oxidized below the aRPD implying biodiffusion, bioadvection, and redox oscillations, were taking place, and though limited, the functions of bioturbation described above were likely balancing and buffering the upper sediment column preventing it from completely shifting to a reduced system. As a result, the influence of infauna bioturbation on sediment geochemistry in CLB was equally as important as the effect of sediment geochemistry on infauna survival and behavior (as represented by bioturbative activity).

## Supporting information

S1 File Raw data. This excel file contains two tabs that compose all of the raw data for the study.
(XLSX)
